# Utility of chemokines CCL2, CXCL8, 10 and 13 and interleukin 6 in the pediatric cohort for the recognition of neuroinflammation and in the context of traditional cerebrospinal fluid neuroinflammatory biomarkers

**DOI:** 10.1371/journal.pone.0219987

**Published:** 2019-07-29

**Authors:** Zuzana Liba, Hana Nohejlova, Vaclav Capek, Pavel Krsek, Anna Sediva, Jana Kayserova

**Affiliations:** 1 Department of Pediatric Neurology, 2nd Faculty of Medicine, Charles University and Motol University Hospital, Prague, Czech Republic; 2 Department of Neurology, 2nd Faculty of Medicine, Charles University and Motol University Hospital, Prague, Czech Republic; 3 Bioinformatics Centre, 2nd Faculty of Medicine, Charles University and Motol University Hospital, Prague, Czech Republic; 4 Department of Immunology, 2 Faculty of Medicine, Charles University and Motol University Hospital, Prague, Czech Republic; Katholieke Universiteit Leuven Rega Institute for Medical Research, BELGIUM

## Abstract

**Background:**

The recognition of active inflammation in the central nervous system (CNS) in the absence of infectious agents is challenging. The present study aimed to determine the diagnostic relevance of five selected chemo/cytokines in the recognition of CNS inflammation and in the context of traditional cerebrospinal fluid (CSF) biomarkers (white blood cell [WBC] counts, oligoclonal bands, protein levels, CSF/serum albumin ratios) and clinical diagnoses.

**Methods:**

C-C and C-X-C motif ligands (CCL2, CXCL8, 10 and 13) and interleukin (IL) 6 levels in the CSF and serum from 37 control and 87 symptomatic children with ten different (mostly noninfectious) inflammatory CNS disorders (16 of which had follow-up samples after recovery) were determined using Luminex multiple bead technology and software. Nonparametric tests were used; p < 0.05 was considered statistically significant. Receiver operating characteristic curves were constructed to analyze controls and 1) all symptomatic samples or 2) symptomatic samples without CSF pleocytosis.

**Results:**

Compared with the control CSF samples, levels of all investigated chemo/cytokines were increased in symptomatic CSF samples, and only IL-6 remained elevated in recovery samples (p ≤ 0.001). CSF CXCL-13 levels (> 10.9 pg/mL) were the best individual discriminatory criterion to differentiate neuroinflammation (specificity/sensitivity: 97/72% and 97/61% for samples without pleocytosis), followed by CSF WBC counts (specificity/sensitivity: 97/62%). The clinical utility of the remaining CSF chemo/cytokine levels was determined in descending order of sensitivities corresponding to thresholds that ensured 97% specificity for neuroinflammation in samples without pleocytosis (pg/mL; sensitivity %): IL-6 (3.8; 34), CXCL8 (32; 26), CXCL10 (317; 24) and CCL2 (387; 10). Different diagnosis-related patterns of CSF chemo/cytokines were observed.

**Conclusions:**

The increased CSF level of CXCL13 was the marker with the greatest predictive utility for the general recognition of neuroinflammation among all of the individually investigated biomarkers. The potential clinical utility of chemo/cytokines in the differential diagnosis of neuroinflammatory diseases was identified.

## Introduction

Neuroimmunological diseases represent a broad spectrum of rare but serious disorders. The recognition of active inflammation in the central nervous system (CNS) in the absence of infectious agents is challenging. Currently available cerebrospinal fluid (CSF) or serum biomarkers and magnetic resonance imaging (MRI) have limited sensitivity and specificity, and novel biomarkers of CNS inflammation are constantly being assessed [1–3].

Under neuroinflammatory conditions, circulating immune cells in the peripheral blood gain access to the CNS, and CSF pleocytosis is a crucial hallmark of neuroinflammation [4]. CSF white blood cell (WBC) counts might fluctuate over time and according to disease activity, and in patients with noninfectious inflammatory CNS diseases, CSF pleocytosis might lack sensitivity [5–7].

Both animal and human studies show that chemokines play an important role in (neuro)inflammation, as chemokines and their corresponding receptors are required for leukocyte migration and function [8–12]. Glial cells, neurons, endothelial cells and immune cells themselves are capable intrathecal chemokine producers [13–16].

Certain C-C and C-X-C motif ligand (CCL and CXCL, respectively) chemokines are frequently investigated in patients with CNS disorders of different etiologies, but their clinical utility has yet to be clearly established [17]. CXCL13, one of the most commonly studied chemokines in neuroinflammation, is a crucial chemokine for B-cell recruitment to the CNS [18]. Increased intrathecal CXCL13 production has been observed in patients with multiple sclerosis (MS) and other noninfectious CNS disorders, and strikingly in neuroborreliosis (NB) [16–22]. CXCL10 is one of several chemokines that mediates T-cell migration and plays an important role in neuroinflammatory models [10, 14]. Elevated intrathecal CXCL10 production has been noted in patients with infectious and noninfectious encephalitis, as well as in patients with MS [22–28]. CXCL8 (known as interleukin [IL] 8) plays a key role in neutrophil transmigration, and CCL2 (known as monocyte chemoattractant protein [MCP] 1) is one of the chemokines involved in controlling monocytes/macrophage and dendritic cell migration. Nonredundant functions have been described for these chemokines during neuroinflammation in animal models [15, 29]. Increased intrathecal CXCL8/IL-8 and CCL2/MCP-1 levels have been found in patients with infectious, particularly bacterial, CNS disorders [30, 31]. To date, the few studies that have investigated CXCL8/IL-8 and CCL2/MCP-1 levels in patients with noninfectious inflammatory CNS disorders have produced inconsistent results [27, 28, 32–36].

In addition to chemokines, IL-6, a pleiotropic cytokine with contradictory proinflammatory and neuroprotective functions, has also been frequently investigated in the context of neuroinflammation. Increased CSF IL-6 levels have been observed in patients with infectious meningitis and some noninfectious inflammatory CNS disorders, but not in patients with MS [30, 37].

In the present study, we investigated the clinical utility of CCL2/MCP-1, CXCL8/IL-8, CXCL10, CXCL13, and IL-6 in the general recognition of neuroinflammation by evaluating their CSF and serum levels in a large cohort of pediatric patients with various (mostly noninfectious) inflammatory CNS disorders. We also compared these markers to traditional CSF neuroinflammatory biomarkers, such as WBC counts, oligoclonal bands (OCBs) or markers of a blood-brain barrier (BBB) failure. Finally, we outlined “disease-specific chemo/cytokine patterns” for selected diagnoses using multiparametric visualization tools.

## Patients and methods

### Ethics statement

This study was approved by the Ethics Committee at Motol University Hospital. Informed written consent to a detection of chemo/cytokine levels in CSF and serum was obtained from parents or guardians of all pediatric participants. The data were analyzed anonymously.

### Study design

**Fig 1:**
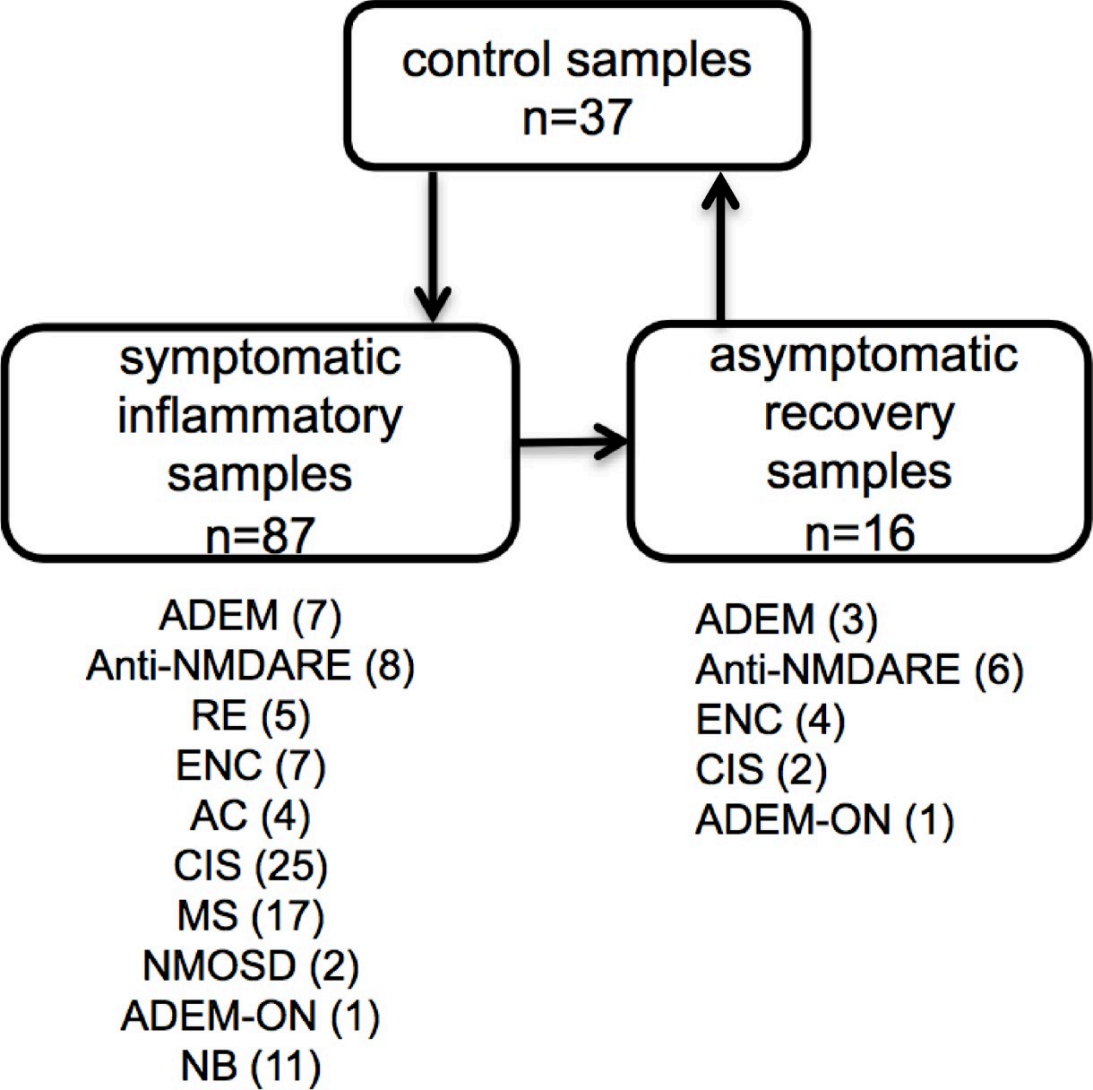
Study group description and design. CSF and serum samples from patients with various, mostly noninfectious, inflammatory CNS disorders collected at the time of presentation of clinical symptoms (n = 87) were compared with controls (n = 37). If available, follow-up samples collected at the time of clinical recovery (n = 16) were compared with their symptomatic counterparts and controls.

In total, 140 pairs of CSF and serum samples from 37 controls and 87 symptomatic children (patients) with various inflammatory CNS disorders (follow-up samples after recovery were obtained from 16 of these patients) were analyzed (Fig 1). All specimens were obtained at the Department of Pediatric Neurology, Charles University, 2nd Faculty of Medicine and Motol University Hospital, Prague, Czech Republic during the routine diagnostic process and/or treatment management.

### Patient characteristics

**Patients** (ethnicity: all Caucasian, with the exception of one Asian; age: median 13 years, range 2–18 years; sex: 61% female) were diagnosed with the following inflammatory CNS disorders: acute disseminated encephalomyelitis (ADEM, n = 7) and ADEM followed by optic neuritis (ADEM-ON, n = 1), anti-N-methyl-D-aspartate receptor encephalitis (NMDARE, n = 8), Rasmussen encephalitis (RE, n = 5), acute cerebellitis of unknown etiology (AC, n = 4), encephalitis of unknown etiology (ENC, n = 7), clinically isolated syndrome (CIS, n = 25), MS (n = 17), neuromyelitis optica spectrum disorders (NMOSD, n = 2) and NB (n = 11). The clinical manifestations of CIS were optic neuritis (n = 15), acute myelitis (n = 2), brainstem syndrome (n = 2), and focal supratentorial syndrome (n = 2). The clinical manifestations of NB were meningitis (n = 8), meningoradiculitis (n = 1), meningoencephalitis (n = 1), and meningomyeloencephalitis (n = 1).

**Symptomatic inflammatory samples** were collected from all patients at the time of presentation of their clinical symptoms, which were 1) acute (n = 78, duration < 3 months, median 10 days, range 1–90 days), 2) progressive (n = 4, duration > 3 months, median 8 months, range 6–12 months), and 3) relapsing (n = 5, patients who had previously been diagnosed with an inflammatory neurological condition, median 12 days, range 4–28 days). The majority of the symptomatic samples were collected before immunotherapy (72 of 87). The immunotherapy in 11 of 15 patients before sampling included steroids (oral or intravenous) and intravenous immunoglobulins (alone or in combination with steroids); three patients were treated with azathioprine and one with cyclosporine. Follow-up **asymptomatic recovery samples** were available from 16 of 87 patients. The absence of the initial and any new clinical symptoms defined a recovery. The time at which recovery samples were collected differed from patient to patient and diagnosis, and the median was 5 months after onset of the first disease symptoms (range 7 days to 2 years).

**Controls** (ethnicity: all Caucasian; age: median 11 years, range 2–18 years; sex: 62% females) were children with various symptoms for which neuroinflammation was initially considered, but ultimately excluded. Detailed examinations of these children also excluded a neurodegenerative etiology for their symptoms, and CSF, blood and MRI findings were within the normal range. The symptoms and/or final diagnoses were: headache (n = 13), different ophthalmologic impairments (n = 9; macular degeneration, strabismus or transient oculomotor disturbances, or nonorganic visual impairment), different psychological/psychiatric manifestations (n = 13; anxiety, depression, fatigue, phobic vertigo, abnormal nonorganic gait or sensorimotor disturbances, or tics), pavor nocturnus (n = 1), and trauma of the plexus brachialis (n = 1).

### Diagnostic procedures

All children in the study underwent an MRI, lumbar puncture and blood tests. Other specific examinations necessary for diagnosis were indicated in individual cases but those are not relevant for the current study.

A routine analysis of CSF included a determination of the following clinically accepted traditional biomarkers: WBC count, OCB level via isoelectric focusing of immunoglobulin (Ig) G, protein levels, and CSF/serum albumin ratio (Qalb = CSF albumin [mg/L] x 10^3^/ serum albumin [mg/L]). CSF findings of a WBC count < 5 x 10^6^ cells/L, OCB of 0–1, protein levels < 400 mg/L, and Qalb < 5 were considered normal (Table 1, details in S1A Table) [2, 38]. Detailed microbiological testing was performed; appropriate combinations of serological, PCR and cultivation methods were used to reveal the presence of pathogens in CSF and/or blood. Tests were performed for common agents causing infectious CNS inflammations in our region, such as herpetic viruses, enteroviruses, virus of tick-borne encephalitis, *Borrelia* spp., mycoplasma pneumonia, etc. If indicated, immunological tests for the presence of anti-NMDAR and other neuropil antibodies, anti-aquaporin 4 (AQP4) and occasionally anti-myelin-oligodendrocyte glycoprotein (MOG) IgG antibodies were performed using commercial kits based on indirect immunofluorescence technique (anti-glutamate receptor [type NMDA] or autoimmune encephalitis mosaic 1 [glutamate receptors type NMDA and AMPA, LGI1, CASPR2, GABA_B_ receptors antibodies], anti-AQP4, anti- MOG, IIFT, Euroimmun, Germany).

**Table 1:** Clinical and laboratory characteristics of the samples.

All samples	Symptomatic inflammatory	Asymptomatic recovery*	Controls
	n = 87	n = 16	n = 37
**Age (years), median (range)**	13 (2–18)	13 (3–19)	11 (2–18)
**Females, n (%)**	53 (61%)	11 (69%)	23 (62%)
**Symptoms: acute/progressive/relapse** ^**#**^	78/4/5	N/A	N/A
**No ImmunoTx, n (%)**	72 (83%)	7 (44%)	37 (100%)
**CSF pleocytosis (> 5 x 10**^**6**^ **cells/L), n (%)**	35 (40%)	1 (6%)	0
**CSF WBC count, median (range) x 10**^**6**^ **cells/L**	3.3 (0–693)	0.2 (0–11)	0.3 (0–2)
**CSF IgG OCB positive (> 2), n (%)**	33 (38%)	2 (13%)	0
**CSF protein, median (range), mg/dL**	0.307 (0.123–1.955)	0.241 (0.154–0.432)	0.191 (0.105–0.303)
**CSF/serum albumin ratio** ^**§**^**, median (range)**	4.3 (1.5–28)	3.3 (2.7–8.9)	3.1 (1.5–4.7)

* 16 of 87 children had follow-up samples after recovery

^#^ Duration/character of the clinical symptoms: acute (onset of neurological symptoms < 3 months), progressive (neurological symptoms in progression > 3 months), relapse (new neurological symptom in a patient who had been previously diagnosed with an inflammatory neurological condition < 1 month)

^§^ Qalb = CSF albumin [mg/L] x 10^3^/serum albumin [mg/L]

All children with ADEM, ADEM-ON, CIS, and MS met the recent diagnostic consensus criteria of the International Pediatric MS Study Group [39]. All patients with NMDARE presented with typical neuropsychiatric symptoms and nonparaneoplastic production of anti-NMDAR antibodies in the CSF [40]. Patients with RE met Bien’s diagnostic criteria [41]. Children diagnosed with ENC of unknown etiology fulfilled the consensus criteria for encephalitis, which is defined as encephalopathy plus two or more of the following symptoms: fever, seizures, focal neurological deficit, and abnormal laboratory findings compatible with encephalitis (in CSF, on the electroencephalogram or MRI) [42]. Microbiological CSF testing and specific autoantibodies were negative in all of the patients with ENC, but specific antibodies against mycoplasma pneumonia were positive in the serum samples from two patients. Children diagnosed with AC of unknown etiology manifested acute cerebellar syndrome with abnormal findings in the CSF but had negative microbiological test results and normal brain MRI results. Both patients with NMOSD fulfilled Wingerchuck’s 2015 diagnostic criteria and were negative for anti-AQP4 antibodies [43]. All patients with NB had abnormal CSF findings and a positive *Borrelia*-specific CSF/serum antibody index [44].

### Chemokine and cytokine detection

Aliquots of centrifuged CSF and serum samples were immediately stored at -30°C and thawed prior to use for chemo/cytokine analyses.

The concentrations of CCL2/MCP-1, CXCL8/IL-8, CXCL10, CXCL13 and IL-6 were measured using Luminex multiple bead technology. We created our own multiplex panel by combining multiple simplex kits with a basic kit according to the manufacturer’s instructions (MCP-1 Human ProcartaPlex^TM^ Simplex Kit [EXP01B-10281-901], IL-8 Human ProcartaPlex Simplex Kit [EXP01A-10204-901], IP-10 Human ProcartaPlex Simplex Kit [EXP01A-10284-901], BLC Human ProcartaPlex Simplex Kit [EXP01A-12147-901], IL-6 Human ProcartaPlex^TM^ Simplex Kit [EXP01A-10213-901] and ProcartaPlex Human Basic Kit [EXP010-10420-901], ThermoFisher Scientific/former eBioscience, San Diego, CA, USA). Our multiplex panel also included other cytokines that are associated with specific immune responses, lymphocytes functions, and immunoregulation (IL-4, -7, -10, -15, -17A and interferon gamma [IFN g]). The methodological details including assay protocol, standards and sensitivity are available at the manufacturer’s website, http://www.thermofisher.com. All samples were measured undiluted and in doublets. The chemo/cytokine standards were assayed in the same manner as patient samples. The data were collected using a Luminex-100 system (Luminex, Austin, TX, USA).

### Data analysis and statistics

Statistical analyses were performed using R software version 3.4.4 [45]. Graphs were created using GraphPad PRISM software version 6.0 (GraphPad Software, La Jolla, CA, USA). Due to the nature of the data, nonparametric tests were used. The Mann-Whitney U-test was used for unpaired comparisons of CSF or serum samples from controls and patients. The Wilcoxon signed-rank test was used to compare paired CSF and serum samples from symptomatic and recovered patients. Correlations between parameters were determined by calculating the Spearman correlation coefficient. P < 0.05 was considered statistically significant.

The predictive accuracies of biomarkers (traditional biomarkers, chemo/cytokines, and selected combinations) were determined using receiver operating characteristic (ROC) curves and by measuring the area under the ROC curve (AUC). ROC curves for combinations of biomarkers were constructed based on predictive models using 50% of patients; the remaining 50% of patients were used as a test group. The thresholds that provided an optimal trade-off between specificity and sensitivity as a criterion for discriminating CNS inflammatory processes were calculated (i.e., optimal thresholds). In addition to the optimal thresholds, the 97% specificity thresholds and their corresponding sensitivities were also derived from the ROC curves for each CSF chemo/cytokine (i.e., values that ensured an at least 97% specificity and less than 3% false positivity for the CNS inflammatory process).

## Results

### Chemo/cytokine levels under neuroinflammatory and recovery conditions

Chemokines CCL2/MCP-1, CXCL8/IL-8, CXCL10, CXCL13 and cytokine IL-6 were detected in the majority of the patients’ samples. Compared with controls, levels of these chemo/cytokines were significantly increased in symptomatic inflammatory CSF samples (all p < 0.001, Table 2A). In contrast to CSF, symptomatic inflammatory serum samples did not exhibit significant differences, with the exception of decreased CCL2/MCP-1 levels (p = 0.017, Table 2B). Other investigated cytokine levels (IL-4, -7, -10, -15, -17A and IFN g)were below the detection limits in the majority of patients’ samples and thus they were not further analyzed in this study.

**Table 2:** Chemo/Cytokine levels in symptomatic inflammatory samples and controls.

	Samples from symptomatic patients (n = 87)	Controls (n = 37)	Mann-Whitney test
	median (IQR) [pg/mL]	median (IQR) [pg/mL]	
**(A) CSF**			
**CCL2/MCP-1**	165.5 (84.5–238.1)	73.3 (57.5–130.9)	p = 0.0003
**CXCL8/IL-8**	23.0 (10.4–40.4)	6.0 (4.8–12.8)	p < 0.0001
**CXCL10**	150.8 (74.0–504.8)	67.8 (35.3–135.8)	p < 0.0001
**CXCL13**	25.9 (8.1–165.9)	6.0 (3.4–7.2)	p < 0.0001
**IL-6**	1.7 (0.0–12.3)	0.0 (0.0–1.2)	p < 0.0001
**(B) Serum**			
**CCL2/MCP-1**	17.5 (6.7–31.7)	33.3 (9.9–47.3)	p = 0.017
**CXCL8/IL-8**	1.3 (0.0–6.5)	3.1 (0.0–7.3)	NS
**CXCL10**	9.6 (4.6–15.5)	9.6 (6.4–19.1)	NS
**CXCL13**	68.3 (50.0–112.3)	71.3 (52.2–94.2)	NS
**IL-6**	0.0 (0.0–1.4)	0.0 (0.0–0.9)	NS

Samples from all symptomatic patients with inflammatory conditions were compared with controls. The medians, interquartile ranges (IQRs), and statistical significance are shown separately for (A) cerebrospinal fluid and (B) serum levels.

**Fig 2:**
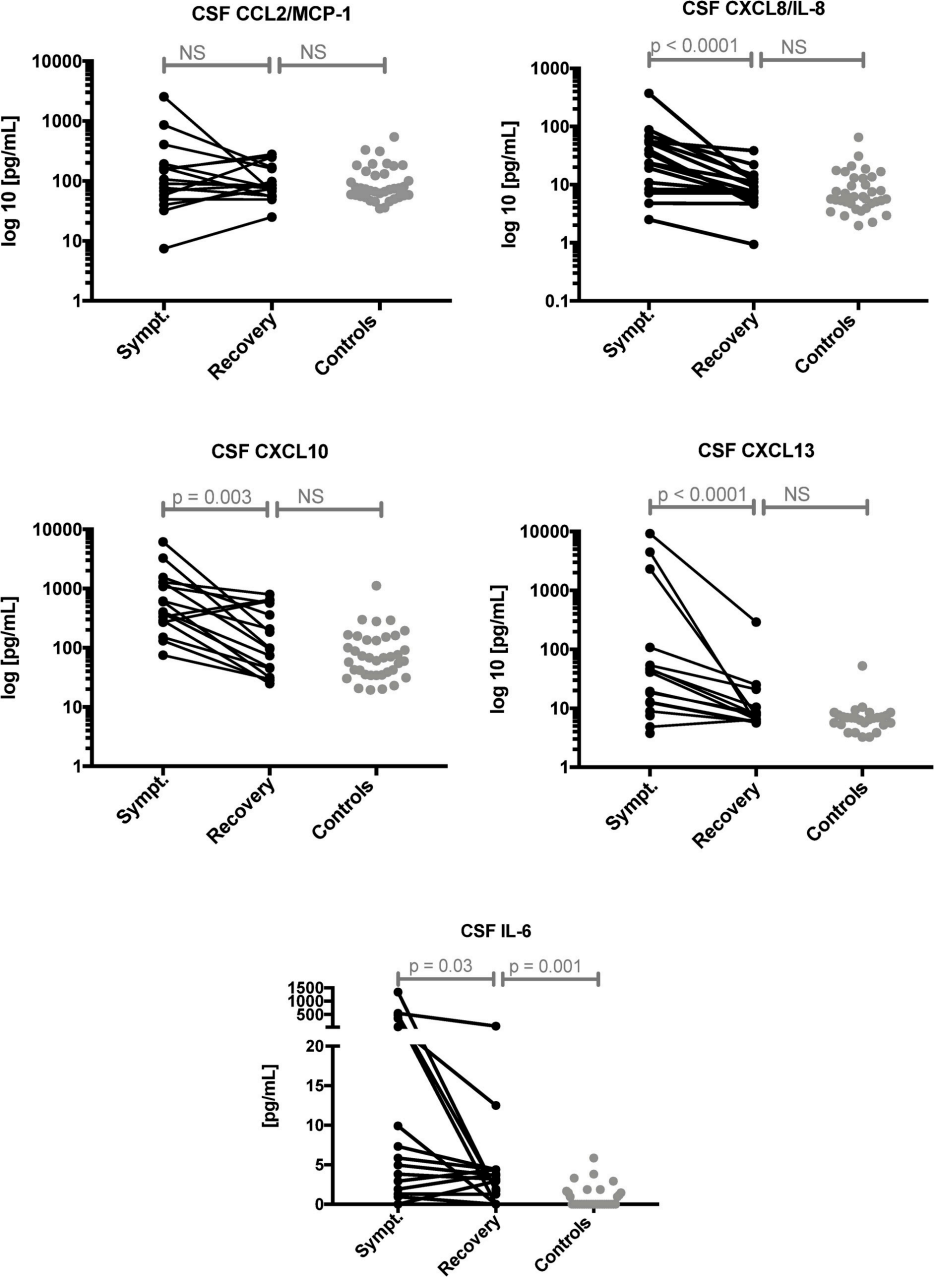
Comparison of CSF chemo/cytokine levels in symptomatic, recovery and control samples. Comparison of chemo/cytokine levels in paired symptomatic and recovery samples (n = 16) using the Wilcoxon signed-rank test and comparisons between recovery samples (n = 16) and controls (n = 37) using unpaired Mann-Whitney tests are displayed, the statistical significance is indicated.

In comparisons of symptomatic and recovery CSF samples, levels of CXCL8/IL-8, CXCL10, CXCL13, and IL-6 were significantly decreased in recovery CSF samples (all p < 0.05) and CCL2/MCP-1 levels showed no significant differences (Fig 2). In recovery sera samples, only CCL2/MCP-1 levels were significantly increased (p = 0.039, S1 Fig). In comparisons of samples from patients who recovered and controls, only IL-6 levels were persistently increased in recovery CSF samples (p = 0.001, Fig 2), while serum chemo/cytokine levels showed no significant differences (S1 Fig).

### Relationship between CSF chemo/cytokines levels and traditional CSF inflammatory biomarkers

The WBC counts, protein levels and CSF/serum albumin ratios in symptomatic inflammatory samples correlated with all CSF chemo/cytokine levels, except for CCL2/MCP-1. The strongest correlation with WBC counts was observed for CXCL13 (r = 0.6459, p < 0.0001). The presence of OCBs in the symptomatic inflammatory samples correlated with CSF CXCL13 and IL-6 levels (S2 Table).

### Clinical utility of chemo/cytokine levels in recognizing CNS inflammation compared with traditional CSF biomarkers

Traditional and chemo/cytokine biomarkers were evaluated individually and in selected combinations. ROC analyses using all symptomatic samples and controls were performed.

For the traditional biomarkers, the individual AUCs were all greater than 0.75, and the optimal thresholds showed a higher specificity (range 78–97%) than sensitivity (50–72%) for differentiating CNS inflammation. The best individual discriminative criterion among the traditional biomarkers was the WBC count, with an optimal threshold of 2.165 x 10^6^ cells/L, yielding a specificity of 97% and sensitivity of 62%. The combination of all four traditional biomarkers improved the predictive accuracy of the test by increasing the specificity to 100% and sensitivity to 87% (Table 3).

**Table 3:** Clinical utility of traditional biomarkers for the recognition of neuroinflammation in CSF.

Traditional CSF biomarkers	AUC	Optimal threshold	Specificity (%)	Sensitivity (%)
**WBC count x 10**^**6**^ **cells/L**	**0.853**	**2.165**	**97**	**62**
**IgG OCB**	0.750	0.5	100	50
**Protein [mg/dL]**	0.790	0.227	78	72
**CSF/serum albumin ratio**	0.780	3.9	88	61
**Combination of all biomarkers**	**0.954**	N/A	100	87

ROC curves were used to determine the clinical utility for the clinically accepted traditional biomarkers of neuroinflammation. All inflammatory samples from the symptomatic patients (n = 87) were analyzed against the controls (n = 37).

**Optimal threshold** shows the value that ensures an optimal trade-off between specificity and sensitivity as a criterion for discriminating CNS inflammatory processes and is determined by AUC; AUC represents the percentage of randomly drawn pairs for which the test is correct (i.e. it truly differentiates between the inflammatory and the control sample).

The individual AUCs for the CSF chemo/cytokine levels were all greater than 0.70. The best discriminative criterion among investigated chemo/cytokines for differentiating CNS inflammation was the CXCL13 level, with an optimal threshold of 10.9 pg/mL; compared with WBC counts, the corresponding specificity was similar (97%), and the sensitivity was higher (72%). In contrast to the traditional biomarkers and CXCL-13 levels, the optimal thresholds for the remaining chemo/cytokines showed lower specificity (range 59–68%) than sensitivity (71–84%). Combination of two chemo/cytokines with the highest individual AUCs (CXCL13 and CXCL8/IL-8) did not reach the individual specificity of CXCL13, but did increase the sensitivity of the test to 86%. Determination of the 97% specificity threshold generated a cut-off value for each chemo/cytokine that ensured a high probability (≥ 97%) of detecting the CNS inflammatory process, but modified a sensitivity. Equalizing of the specificity enabled to compare corresponding sensitivities and assess the clinical utility of the remaining chemo/cytokines for the recognition of CNS inflammation as follows: IL-6 (sensitivity 40%), CXCL10 (38%), CXCL8/IL-8 (31%) and CCL2/MCP-1 (12%) (Table 4A).

**Table 4:** Clinical utility of CSF chemo/cytokine biomarkers for the recognition of neuroinflammation.

**(A) All inflammatory samples (n = 87)**						
**CSF chemo/cytokines**	**AUC**	**Optimal threshold [pg/mL]**	**Specificity (%)**	**Sensitivity (%)**	97% specificity threshold [pg/mL]^*^	***Sensitivity***^***#***^ ***(%)***
**CCL2/MCP-1**	0.703	81.4	65	77	*339*.*5*	*12*
**CXCL8/IL-8**	0.797	8.0	67	84	*30*.*9*	*31*
**CXCL10**	0.735	7.5	59	79	*307*.*5*	*38*
**CXCL13**	**0.866**	**10.9**	**97**	**72**	***10*.*9***	***72***
**IL-6**	0.732	0.3	68	71	*3*	*40*
**CXCL8/IL-8 + CXCL13**	0.885	N/A	94	86	*N/A*	*N/A*
**Combination of all biomarkers**	0.916	N/A	89	84	*N/A*	*N/A*
**(B) Inflammatory samples without CSF pleocytosis (n = 52)**						
**CSF chemo/cytokines**	**AUC**	**Optimal threshold [pg/mL]**	**Specificity (%)**	**Sensitivity (%)**	97% specificity threshold [pg/mL]^*^	***Sensitivity (%)***^***#***^
**CCL2/MCP-1**	0.728	94.9	68	80	*386*.*7*	*10*
**CXCL8/IL-8**	0.827	8.0	68	90	*32*.*4*	*26*
**CXCL10**	0.649	67.9	54	76	*317*.*4*	*24*
**CXCL13**	**0.805**	**7.6**	**79**	**80**	***10*.*9***	***61***
**IL-6**	0.737	0.3	68	72	*3*.*8*	*34*
**CXCL8 + CXCL13**	0.875	N/A	94	83	*N/A*	*N/A*
**Combination of all biomarkers**	0.951	N/A	94	88	*N/A*	*N/A*

ROC curves were used to determine the clinical utility for the investigated chemo/cytokine levels. Two different groups of patients’ samples were analyzed against controls (n = 37): (A) all inflammatory samples from symptomatic patients (n = 87) and (B) only inflammatory samples from symptomatic patients without CSF pleocytosis (i.e. with CSF WBC counts < 5 x 10^6^ cells/L, n = 52).

**Optimal threshold** shows the value that ensures an optimal trade-off between specificity and sensitivity as a criterion for discriminating CNS inflammatory processes and is determined by AUC; AUC represents the percentage of randomly drawn pairs for which the test is correct (i.e. it truly differentiates between the inflammatory and the control sample).

^*^97% specificity threshold shows the value that ensures a high probability (≥ 97%) of a truly recognition of an inflammatory sample, if the particular level of the chemo/cytokine in a sample exceeds this value.

^*#*^***sensitivity*** modified sensitivity corresponding to the 97% specificity threshold

The individual AUCs for serum chemo/cytokines levels did not exceed 0.64, which was the AUC for CCL2/MCP-1, yielding 62% specificity and 69% sensitivity (S3 Table).

### CSF chemo/cytokine levels and their clinical utility for recognizing CNS inflammation in the absence of CSF pleocytosis

In 52 symptomatic inflammatory samples (60%), the WBC counts were within the clinically accepted normal range (< 5 x 10^6^/L). Moreover, in 23/52 samples, the other traditional CSF biomarkers also showed no noticeable abnormalities. In addition, no infectious agents were detected in these CSF samples. Thus, we performed a separate analysis of this subgroup; the optimal and 97% specificity thresholds were determined by ROC analyses using only symptomatic inflammatory CSF samples without pleocytosis and controls.

The individual AUCs for the CSF chemo/cytokine levels in samples from patients without pleocytosis were all greater than 0.65 and optimal thresholds showed a lower specificity (range 54–79%) than sensitivity (72–90%) for differentiating neuroinflammation. According the AUC, CXCL8/IL-8 was the best discriminative criterion in this subgroup, followed by CXCL13. However, in contrast to CXCL13, the optimal threshold for CXCL8/IL-8 yielded a lower specificity (68% vs. 79%). Combinations of all five chemo/cytokines or the best two (CXCL8/IL-8 and CXCL13) improved the predictive accuracies of the test by increasing specificity to 94% and sensitivity up to 88%. The use of the 97% specificity threshold and its corresponding sensitivity helped us to assess the clinical utility for each chemo/cytokine for the recognition of neuroinflammation in patients without CSF pleocytosis as follows: CXCL13 (sensitivity 61%), IL-6 (34%), CXCL8/IL-8 (26%), CXCL10 (24%) and CCL2/MCP-1 (10%) (Table 4B).

### CSF chemo/cytokines levels according to diagnosis

**Fig 3:**
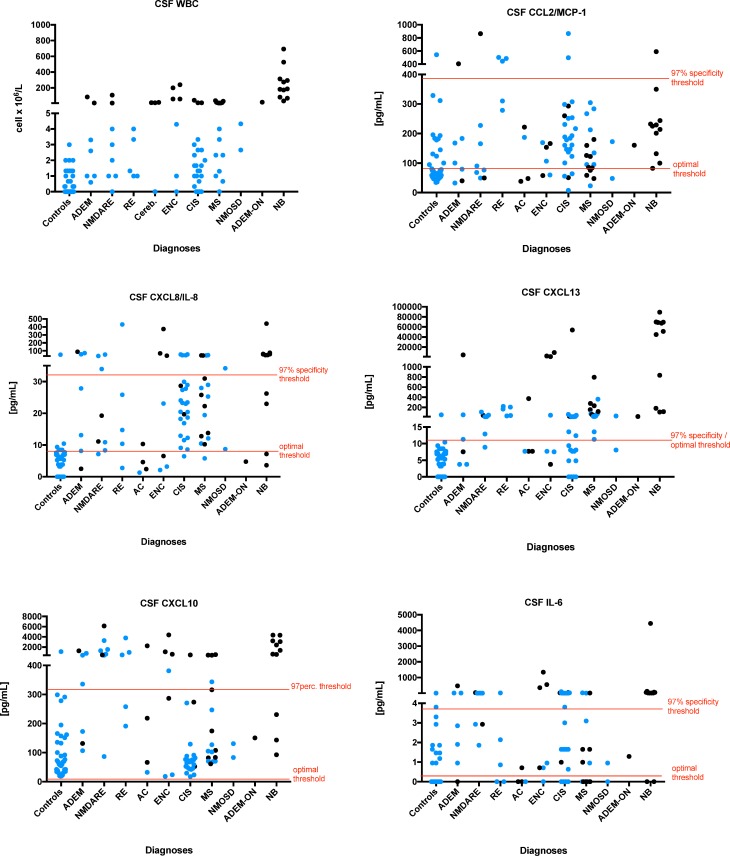
Pleocytosis and chemo/cytokine levels in the CSF according to the diagnosis. The blue dots indicate samples from patients without CSF pleocytosis (< 5 cells/μL). The lines in graphs of CSF chemo/cytokine levels indicate values of 1) optimal thresholds determined by ROC analyses using all symptomatic inflammatory samples and controls and 2) 97% specificity thresholds derived from ROC curves using only symptomatic inflammatory samples without pleocytosis and controls. The optimal and 97% specificity thresholds are identical for CXCL13.

When we focused on CSF chemo/cytokines levels in the context of diagnoses and 97% specificity thresholds (derived from the ROC curve for samples without pleocytosis), we noted certain diagnosis-related differences (Fig 3). CXCL13 levels were increased in patients with all diagnoses, but exceeded the threshold (10.9 pg/mL) in all CSF samples from patients with NB, RE and MS. Interestingly, CXCL10 levels exceeded the threshold (317 pg/mL) in 82% (9/11) of patients with NB and in 66% (18/27) of patients with encephalitis (ADEM, NMDARE, RE, or ENC) but only in 24% (5/17) of patients with MS and in 4% (1/25) of patients with CIS. Furthermore, IL-6 levels exceeded the threshold (3.8 pg/mL) in 82% (9/11) of patients with NB and in 44% (11/25) of patients with CIS, but only in 18% (3/17) of patients with MS. Additional diagnosis-related details regarding CSF chemo/cytokines levels are summarized in S1B Table.

**Fig 4:**
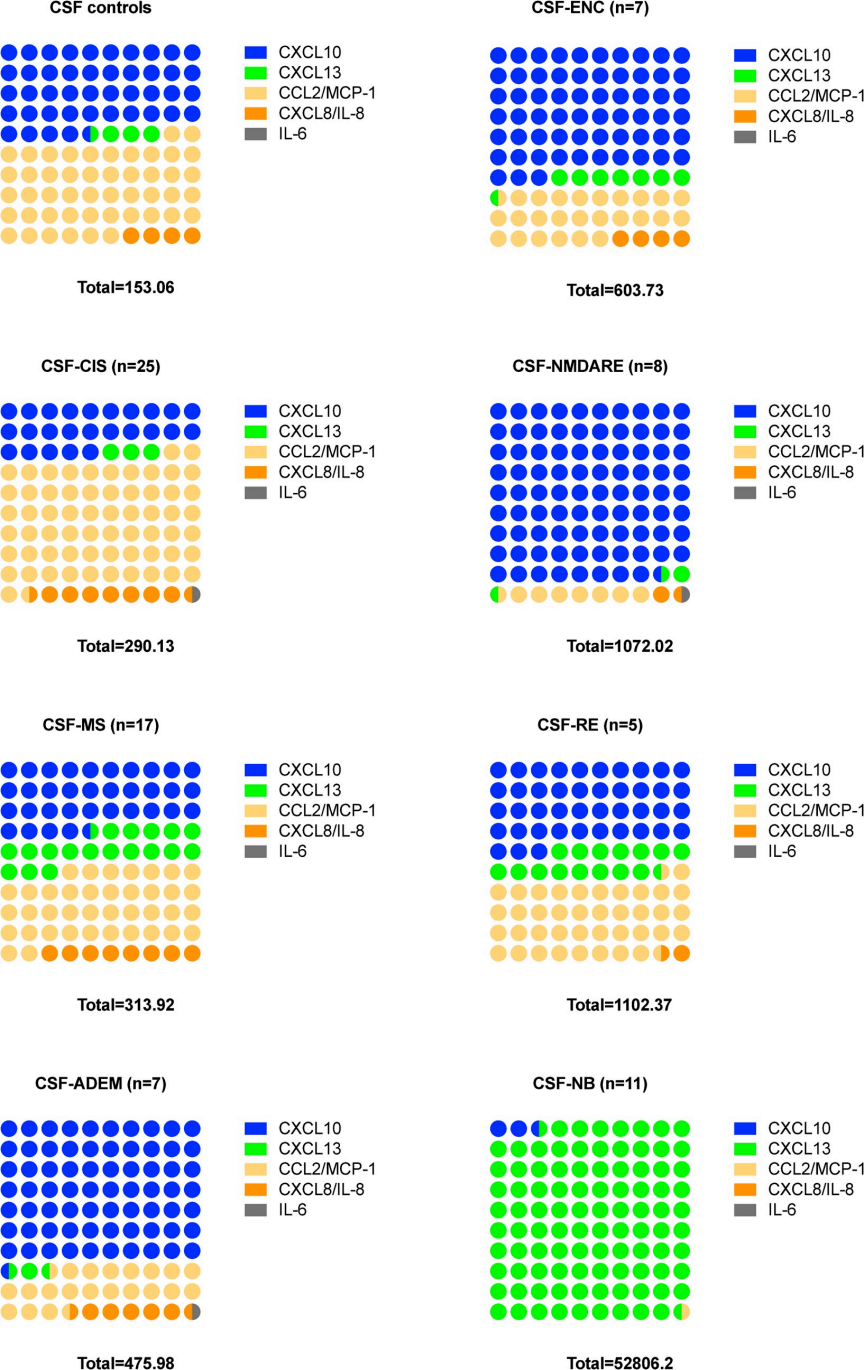
Diagnosis-related chemo/cytokine patterns. Differences in the proportional and quantitative involvement of the investigated chemo/cytokines in patients with different diagnoses, for which at least five CSF samples were available, are shown in multiparametric graphs. Median values for particular CSF chemo/cytokine levels were used; total number under each graph is the sum of these medians in the particular diagnosis.

Finally, we created multiparametric graphs and displayed the sum of medians of investigated chemo/cytokine levels for those diagnoses for which at least five CSF samples each were available. Thus, we revealed the different proportional and quantitative chemo/cytokines involvement in each diagnosis (Fig 4). NB and NMDARE were two diagnoses in which the markedly different proportions of involved chemo/cytokines was best exemplified; CXCL13 dominated in NB, while CXCL10 dominated in NMDARE. Moreover, regarding the sum of the medians of investigated chemo/cytokines in certain diagnosis, we observed the highest total value in patients with NB (52,806). In noninfectious diagnoses these total values were markedly lower (range 290–1,102), but higher in patients with encephalitis (particularly RE and NMDARE) than in patients with demyelinating disorders (MS or CIS).

## Discussion

Chemo/cytokines play a substantial role in neuroinflammation [46]. However, heterogeneity, variability and inconsistencies in many previous studies have hampered to corroborate results, and thus constrain the clinical utility of these markers [47].

In contrast to many previous studies, we did not primarily focus on a particular disease or group of diseases, such as MS [32–36]. We tested the clinical relevance of four potent chemoattractants of the major leukocyte subtypes and one pluripotent cytokine for the general recognition of CNS inflammation and in the context of traditional biomarkers. CSF levels of the selected chemo/cytokines are frequently increased, particularly in patients (either adult or pediatric) with infectious diseases [26, 30, 31, 47–50]. The selected chemo/cytokines were detected in the majority of symptomatic patients‘ samples in our study. Despite the majority of them were obtained from patients with noninfectious inflammatory CNS disorders, CSF levels of all investigated chemo/cytokine in these patients were also higher than in controls, while in serum minimal differences were noticed. The levels of some chemo/cytokines have also been shown to decrease after successful treatment or recovery [19, 49, 51]. In our study, CSF levels of all chemokines (but not IL-6) were decreased in asymptomatic patients. Due to the variety of diagnoses, only the presence of neurological symptoms at the time of CSF sampling, but not the disease duration or treatment, was considered in our analyses.

The proper evaluation of chemo/cytokine levels critically depends on the controls [52]. Individual chemo/cytokine reference ranges have not yet been clearly established. The Pranzatelli group demonstrated the compartmentalization of chemo/cytokines and higher concentrations of CXCL10 and CCL2/MCP-1 than CXCL8/IL-8 and IL-6 in the CSF of pediatric controls [53]. In addition, other researchers have also observed low CSF CXCL13 levels in controls [28, 54]. We used different assays than recent pediatric studies, and our controls were patients without neurological disorders. Our conclusions were similar to those reported in previous studies, but the absolute ranges of values for the controls differed, particularly the CXCL8/IL-8, CXCL10 and CCL2/MCP-1 levels, for which our upper limits in the controls were lower [28, 53].

Clinicians need reliable markers and cut-off values that indicate neuroinflammatory conditions with a high probability [55]. In some studies, the 95^th^ percentile of the control values was used as a cut-off to analyze patient samples [28]. A ROC analysis is a useful diagnostic test that enables the determination of an optimal trade-off between the specificity (true positivity) and sensitivity (true negativity) of a tested marker by classifying the two subjects in the pair according the clinical task [56]. Thus, depending on the study design, the absolute threshold value for the same marker can vary. In our study, we asked how precisely the selected biomarkers differentiated neuroinflammatory samples from controls. We also hypothesized that the detection of chemo/cytokine levels would be especially useful in situations where traditional biomarkers were negative, and thus we individually tested a subgroup of samples from patients without pleocytosis. In addition to the optimal thresholds, we also used the ROC curves to derive threshold values with high specificity for neuroinflammation (i.e., 97% specificity thresholds), which helped us to more accurately assess the clinical utility of individual chemo/cytokines.

Taken together, our data generally demonstrate the insufficient sensitivity of currently available traditional CSF biomarkers in discriminating noninfectious inflammatory CNS disorders and highlight CXCL13 as the best individual biomarker of neuroinflammation of various etiology. CXCL13 levels were increased in patients with all diagnoses, and consistent with other reports, CXCL13 levels correlated with the traditional biomarkers [18, 21, 36, 50, 54]. The majority of our analyzed samples were from patient with acute clinical symptoms, but CXCL13 levels were increased also in all samples from patients with progressive neurological symptoms and in 4/5 samples from patients with relapsing symptoms. Other individually investigated chemokines, as well as IL-6, lacked specificity in the CSF. In the absence of CSF pleocytosis, CXCL13 was the marker with the highest individual specificity, but combinations of all chemo/cytokines or the two (CXCL8/IL-8 and CXCL13) augmented the predictive accuracy for differentiating neuroinflammation. According to our data, CCL2/MCP-1 was the poorest predictive biomarker of neuroinflammation. Although this chemokine showed significantly different CSF levels and was the only biomarker to show differences in serum levels in patients, the ROC analysis did not reveal any convincing utility for these findings. This result is consistent with previous reports of the limited clinical utility of CCL2/MCP-1 levels in patients with MS and encephalitis [28, 33].

We had to specifically focus on disease-specific chemo/cytokine CSF levels to better compare our findings with previously published data in pediatric and adult patients. The recent review shows congruent results in certain CNS disorders of either pediatric- or adult-onset [47]. Thus similar patterns of analyzed chemo/cytokines as in our pediatric cohort can be expected in adults. Due to the multivariateble data and the relatively small number of samples for patients with certain diagnoses in our study, we neither performed comparative statistics between diagnoses nor disease-specific ROC analyses. The individually high predictive value of CXCL13 has been observed in patients with NB [50, 57, 58]. CXCL13 and CXCL8/IL-8 have been already studied (individually and in combination with IL-12p40 or CXCL10) in the context of MS and neurosyphilis [36, 49]. Despite the different absolute values, both markers showed high predictive accuracies for the diseases, and consistent with our findings, the corresponding specificity of CXCL8/IL-8 for MS was lower than the sensitivity. Quantitative differences in the concentration of a particular chemo/cytokine are also postulated to be useful as a diagnostic aid in patients with neuroinflammatory disorders [47]. Different individual CSF cut-off values have been proposed for adults, such as 7.7 pg/mL or 15.4 pg/mL for CXCL13 to predict the progression of CIS to definitive MS [54, 59] and 10 pg/mL for IL-6 to exclude MS [37]. Compared with these studies, we observed lower CXCL13 levels in children with CIS than in individuals with MS, and only 2/17 patients with pediatric MS exceeded the proposed cut-off value for IL-6 [37]. Based on accumulating evidence, CSF CXCL10 levels are elevated in patients with encephalitis of either an infectious or noninfectious etiology [27, 28, 60]. The predictive accuracy of CXCL10 in the general recognition of neuroinflammation was low in our study. However, a specific focus on samples from patients with encephalitis revealed increased CXCL10 levels compared with patients with demyelinating disorders or controls.

Using multiparametric graphs, we finally utilized the potential of our data to visualize the different proportional and quantitative involvement of chemo/cytokines in certain diagnoses. All these disease-related findings supported the hypothesized additional clinical utility of the investigated chemo/cytokines in the differential diagnosis of neuroinflammatory conditions. Nevertheless, we are aware that further studies are needed to identify disease-specific chemo/cytokine patterns that may have diagnostic relevance in the future.

## Conclusion

Our study provided unique data on the levels of five chemo/cytokines using the same assay method simultaneously in a pediatric cohort of patients with one of ten different inflammatory (mostly noninfectious) CNS disorders. The increased CSF level of CXCL13 was the biomarker with the greatest predictive utility for the general recognition of neuroinflammation among all of the individually investigated biomarkers. The results of our study also revealed the potential clinical utility of the investigated chemo/cytokines in the differential diagnosis of certain neuroinflammatory diseases.

## Supporting information

S1 Fig Comparison of serum chemo/cytokine levels in symptomatic, recovery and control samples. Comparison of chemo/cytokine levels in paired symptomatic and recovery samples (n = 16) using the Wilcoxon signed-rank test and comparisons between recovery samples (n = 16) and controls (n = 37) using unpaired Mann-Whitney tests are displayed, the statistical significance is indicated.
(TIF)

S1 Table Clinical and laboratory findings in the samples stratified according to the diagnosis. **Table A**. Clinical and laboratory characteristics of the samples stratified according to diagnosis. **Table B**. Number of patients with increased CSF chemo/cytokines levels exceeding the “97% specificity threshold” stratified according to diagnosis.
(DOCX)

S2 Table Correlations between CSF chemo/cytokine levels and traditional CSF biomarkers of neuroinflammation. (DOCX)

S3 Table Clinical utility of serum chemo/cytokine biomarkers for the recognition of neuroinflammation. (DOCX)

S4 Table Raw data. (XLSX)

## Abbreviations

AC: acute cerebellitis
ADEM: acute disseminated encephalomyelitis
AQP4: aquaporine 4
CCL2/MCP-1: C-C motif ligand 2/monocyte chemoattractant protein 1
CIS: clinically isolated syndrome
CSF: cerebrospinal fluid
CXCL8/IL-8: C-X-C motif ligand 8/interleukine 8
CXCL10 and 13: C-X-C motif ligand 10 and 13
ENC: encephalitis
IL-6: interleukine 6
MOG: myelin oligodenrocyte glycoprotein
MRI: magnetic resonance imaging
MS: multiple sclerosis
NB: neuroborreliosis
NMDARE: N-methyl-D-aspartate receptor encephalitis
NMOSD: neuromyelitis optica spectrum disorders
OCB: oligoclonal bands
ON: optic neuritis
Qalb: CSF/serum albumin ratio
RE: Rasmussen encephalitis
ROC: receiver operating characteristic
WBC: white blood cell
