# Supplementary figures and images for: Utility of chemokines CCL2, CXCL8, 10 and 13 and interleukin 6 in the pediatric cohort for the recognition of neuroinflammation and in the context of traditional cerebrospinal fluid neuroinflammatory biomarkers
Source: PLoS One. 2019 Jul 29;14(7):e0219987. doi: 10.1371/journal.pone.0219987 (PMC6663008; doi:10.1371/journal.pone.0219987)

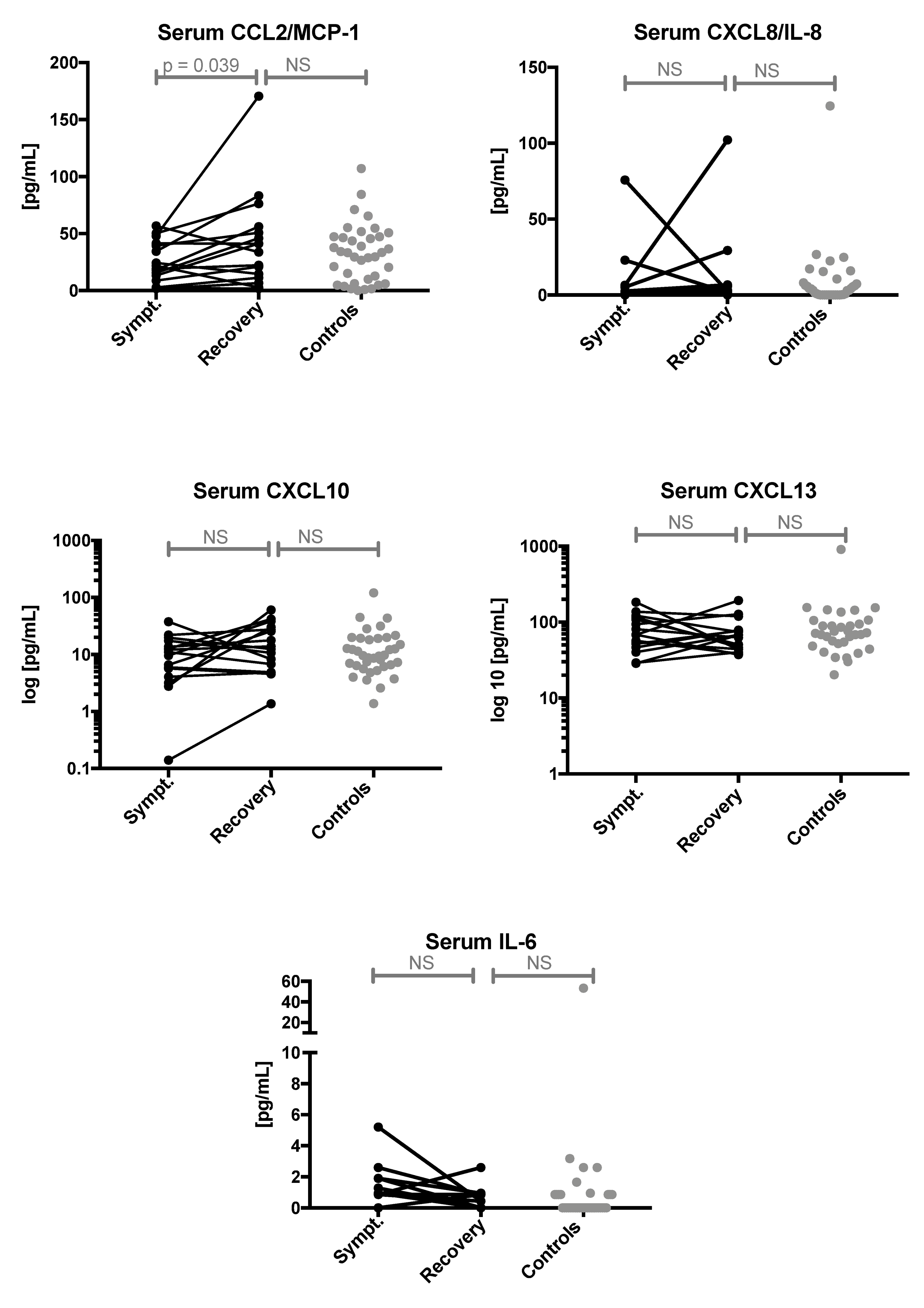

Supplement: S1 Fig — Comparison of chemo/cytokine levels in paired symptomatic and recovery samples (n = 16) using the Wilcoxon signed-rank test and comparisons between recovery samples (n = 16) and controls (n = 37) using unpaired Mann-Whitney tests are displayed, the statistical significance is indicated. (TIF) [file pone.0219987.s001.tif]
