# Supplementary material for: Utility of chemokines CCL2, CXCL8, 10 and 13 and interleukin 6 in the pediatric cohort for the recognition of neuroinflammation and in the context of traditional cerebrospinal fluid neuroinflammatory biomarkers
Source: PLoS One. 2019 Jul 29;14(7):e0219987. doi: 10.1371/journal.pone.0219987 (PMC6663008; doi:10.1371/journal.pone.0219987)
Supplement: S1 Table — Table A. Clinical and laboratory characteristics of the samples stratified according to diagnosis. Table B. Number of patients with increased CSF chemo/cytokines levels exceeding the “97% specificity threshold” stratified according to diagnosis. (DOCX) [file pone.0219987.s002.docx]

**S1 Table: Clinical and laboratory findings in the samples stratified according to the diagnosis**

**A)** Clinical and laboratory characteristics of the samples stratified according to diagnosis

| **Symptomatic samples and diagnoses** | **ADEM** | **NMDARE** | **RE** | **ENC** | **AC** | **CIS** | **MS** | **NMOSD** | **ADEM-ON** | **NB** |
| --- | --- | --- | --- | --- | --- | --- | --- | --- | --- | --- |
|  | **n = 7** | **n = 8** | **n = 5** | **n = 7** | **n = 4** | **n = 25** | **n = 17** | **n = 2** | **n = 1** | **n = 11** |
| **Age (years), median (range)** | 5 (2-15) | 13 (7-19) | 7 (3-10) | 7 (3-18) | 2 (2-12) | 13 (4-17) | 16 (12-18) | 12 (11-15) | 4 | 8 (6-15) |
| **Females, n (%)** | 1 (14%) | 7 (88%) | 1 (80%) | 6 (86%) | 1 (25%) | 15 (60%) | 12 (71%) | 1 (50%) | 1 (100%) | 5 (45%) |
| **Symptoms: acute/progressive/relapse*** | all acute | all acute | 1/4/none | all acute | all acute | all acute | 15/none/2 | all relapse | all relapse | all acute |
| **No ImmunoTx, n (%)** | 3 (43%) | 7 (88%) | 3 (60%) | 7 (100%) | 4 (100%) | 19 (76%) | 17 (100%) | 0 | 1 (100%) | 11 (100%) |
| **CSF pleocytosis (> 5 x 10^6^ cells/L), n (%)** | 2 (29%) | 2 (25%) | 0 | 4 (57%) | 3 (75%) | 3 (12%) | 9 (53%) | 0 | 1 (100%) | 11 (100%) |
| **CSF WBC, median (range) x 10^6^ cells/L** | 2.6 (0.6-84) | 3 (0-108) | 1.2 (1-3.3) | 59 (0-240) | 12 (0-15) | 1 (0-43) | 6.3 (0-38) | 3.5 (3-4) | 18 | 187 (34-693) |
| **CSF IgG OCB positive (> 2), n (%)** | 1 (14%) | 3 (38%) | 3 (60%) | 1 (14%) | 1 (25%) | 5 (20%) | 16 (94%) | 0 | 0 | 3 (27%) |
| **CSF protein, median (range), mg/dL** | 0.250 (0.177-0.802) | 0.232 (0.178-0.310) | 0.310 (0.290-0.445) | 0.367 (0.182-0.767) | 0.161 (0.123-0.299) | 0.233 (0.136 -0.702) | 0.395 (0.148-0.816) | 0.234 (0.231-0.237) | 202 | 0.705 (0.301-1.955) |
| **CSF/serum albumin ratio**, median (range)** | 7.6 (3.2-11.4) | 3.3 (2.5-4) | 4.5 (4.1-7.1) | 5.3 (1.5-13) | 2.5 (1.9-4.6) | 3.9 (2-11) | 5.4 (2-11.5) | 3.4 (3.3-3.5) | 2.8 | 10 (4.5 -28) |

**B)** Number of patients with increased CSF chemo/cytokines levels exceeding the “97% specificity threshold” stratified according to diagnosis

| **Symptomatic samples and diagnoses** | **ADEM** | **NMDARE** | **RE** | **ENC** | **AC** | **CIS** | **MS** | **NMOSD** | **ADEM-ON** | **NB** |
| --- | --- | --- | --- | --- | --- | --- | --- | --- | --- | --- |
|  | **n = 7** | **n = 8** | **n = 5** | **n = 7** | **n = 4** | **n = 25** | **n = 17** | **n = 2** | **n = 1** | **n = 11** |
| **CSF CXCL13 > 10.9 pg/mL, n (%)** | 3 (43%) | 6 (75%) | 5 (100%) | 4 (57%) | 1 (25%) | 12 (48%) | 17 (100%) | 1 (50%) | 1 (100%) | 11 (100%) |
| **CSF IL-6 > 3.8 pg/mL, n (%)** | 3 (43%) | 5 (63%) | 1 (20%) | 3 (43%) | 0 | 11 (44%) | 3 (18%) | 0 | 0 | 9 (82%) |
| **CXCL10 > 317 pg/mL, n (%)** | 4 (57%) | 7 (87%) | 3 (60%) | 4 (57%) | 1 (25%) | 1 (4%) | 5 (29%) | 0 | 0 | 9 (82%) |
| **CXCL8/IL-8 > 32.4 pg/mL, n (%)** | 3 (43%) | 3 (38%) | 1 (20%) | 3 (43%) | 0 | 4 (16%) | 4 (24%) | 1 (50%) | 0 | 6 (55%) |
| **CCL2/MCP-1 > 386.7 pg/mL, n (%)** | 1 (14%) | 1 (13%) | 3 (60%) | 1 (14%) | 0 | 2 (8%) | 0 | 0 | 0 | 1 (9%) |

**Abbreviations:** AC – acute cerebellitis, ADEM – acute disseminated encephalomyelitis, anti-NMDARE – N-methyl-D-aspartate encephalitis, CIS – clinically isolated syndrome, ENC – encephalitis of unknown etiology, MS – multiple sclerosis, NB – neuroborreliosis, NMOSD – neuromyelitis optica spectrum disorders, ON – optic neuritis, RE – Rasmussen encephalitis
