# Supplementary material for: Utility of chemokines CCL2, CXCL8, 10 and 13 and interleukin 6 in the pediatric cohort for the recognition of neuroinflammation and in the context of traditional cerebrospinal fluid neuroinflammatory biomarkers
Source: PLoS One. 2019 Jul 29;14(7):e0219987. doi: 10.1371/journal.pone.0219987 (PMC6663008; doi:10.1371/journal.pone.0219987)
Supplement: S2 Table — (DOCX) [file pone.0219987.s003.docx]

**S2 Table: Correlations between CSF chemo/cytokine levels and traditional CSF biomarkers of neuroinflammation**

|  | **CSF CCL2/MCP-1** | **CSF CXCL8/IL-8** | **CSF CXCL10** | **CSF CXCL13** | **CSF IL-6** |
| --- | --- | --- | --- | --- | --- |
| **CSF WBC count** | NS | 0.2219 (p = 0.0412) | 0.5663 (p < 0.0001) | 0.6459 (p < 0.0001) | 0.2434 (p = 0.0246) |
| **CSF OCB** | NS | NS | NS | 0.3829 (p < 0.0001) | -0.2442 (p = 0.0271) |
| **CSF protein** | NS | 0.5107 (p < 0.0001) | 0.4639 (p < 0.0001) | 0.5524 (p < 0.0001) | 0.2887 (p = 0.0085) |
| **CSF/serum albumin ratio** | NS | 0.4743 (p < 0.0001) | 0.4803 (p < 0.0001) | 0.5273 (p < 0.0001) | 0.2865 (p = 0.0091) |

Spearman’s correlation coefficients and the corresponding statistical significance values are noted in the table.
