# Supplementary material for: Utility of chemokines CCL2, CXCL8, 10 and 13 and interleukin 6 in the pediatric cohort for the recognition of neuroinflammation and in the context of traditional cerebrospinal fluid neuroinflammatory biomarkers
Source: PLoS One. 2019 Jul 29;14(7):e0219987. doi: 10.1371/journal.pone.0219987 (PMC6663008; doi:10.1371/journal.pone.0219987)
Supplement: S3 Table — (DOCX) [file pone.0219987.s004.docx]

**S3 Table**: **Clinical utility of serum chemo/cytokine biomarkers for the recognition of neuroinflammation**

| **Serum chemo/cytokines (all inflammatory samples)** | **AUC** | **Optimal threshold [pg/mł]** | **specificity (%)** | **sensitivity (%)** |
| --- | --- | --- | --- | --- |
| **CCL2/MCP-1** | 0.636 | 26.2 | 62 | 69 |
| **CXCL8/IL-8** | 0.556 | 2.8 | 54 | 63 |
| **CXCL10** | 0.452 | 9.8 | 54 | 49 |
| **CXCL13** | 0.496 | 64.5 | 67 | 47 |
| **IL-6** | 0.466 | 1.0 | 87 | 27 |
| **Combination of all** | 0.632 | N/A | 59 | 74 |
